# Usefulness and caveats of real-world data for research on hypertension and its association with cardiovascular or renal disease in Japan

**DOI:** 10.1038/s41440-024-01875-5

**Published:** 2024-09-11

**Authors:** Michihiro Satoh, Shingo Nakayama, Maya Toyama, Hideaki Hashimoto, Takahisa Murakami, Hirohito Metoki

**Affiliations:** 1 Division of Public Health, Hygiene and Epidemiology, Tohoku Medical and Pharmaceutical University, Sendai, Japan; 2 Department of Preventive Medicine and Epidemiology, Tohoku Medical Megabank Organization, Tohoku University, Sendai, Japan; 3 Department of Pharmacy, Tohoku Medical and Pharmaceutical University Hospital, Sendai, Japan; 4 Division of Nephrology and Endocrinology, Faculty of Medicine, Tohoku Medical and Pharmaceutical University, Sendai, Japan; 5 Department of Nephrology, Self-Defense Forces Sendai Hospital, Sendai, Japan; 6 Division of Aging and Geriatric Dentistry, Department of Rehabilitation Dentistry, Tohoku University Graduate School of Dentistry, Sendai, Japan; 7 Tohoku Institute for Management of Blood Pressure, Sendai, Japan

**Keywords:** Blood pressure, Cardiovascular diseases, Citizen science, Kidney diseases, Hypertension

## Abstract

The role of real-world data, collected from clinical practice rather than clinical trials, has become increasingly important for investigating real-life situations, such as treatment effects. In Japan, evidence on hypertension, cardiovascular diseases, and kidney diseases using real-world data is increasing. These studies are mainly based on “the insurer-based real-world data” collected as electronic records, including data from health check-ups and medical claims such as JMDC database, DeSC database, the Japan Health Insurance Association (JHIA) database, or National Databases of Health Insurance Claims and Specific Health Checkups (NDB). Based on the insurer-based real-world data, traditional but finely stratified associations between hypertension and cardiovascular or kidney diseases can be explored. The insurer-based real-world data are also useful for pharmacoepidemiological studies that capture the distribution and trends of drug prescriptions; combined with annual health check-up data, the effectiveness of drugs can also be examined. Despite the usefulness of insurer-based real-world data collected as electronic records from a wide range of populations, we must be cautious about several points, including issues regarding population uncertainty, the validity of cardiovascular outcomes, the accuracy of blood pressure, traceability, and biases, such as indication and immortal biases. While a large sample size is considered a strength of real-world data, we must keep in mind that it does not overcome the problem of systematic error. This review discusses the usefulness and pitfalls of insurer-based real-world data in Japan through recent examples of Japanese research on hypertension and its association with cardiovascular or kidney disease.

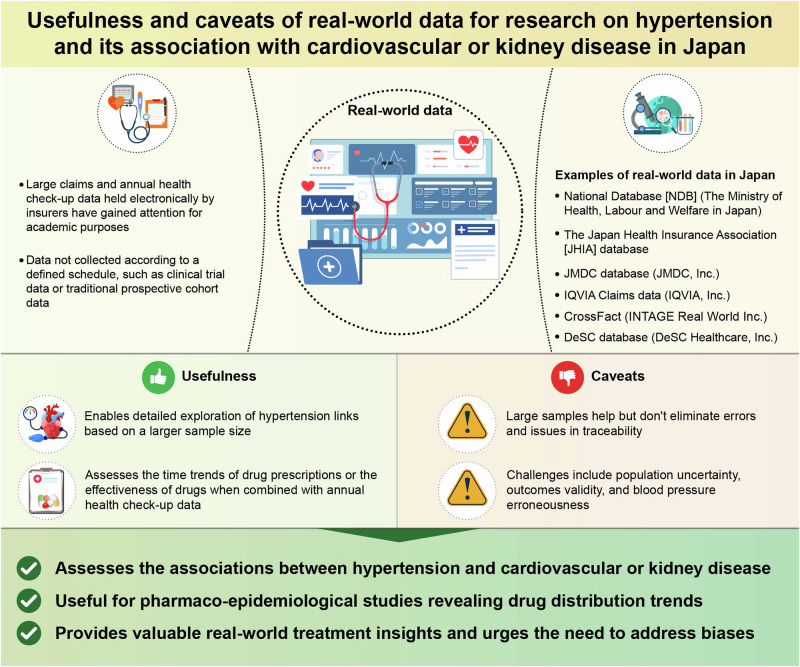

## Introduction

The digitization of medical information has progressed, allowing us to control vast medical or health data. The role of real-world data (RWD) in healthcare has gained importance for investigating real health conditions in both general and patient populations, longitudinal associations between exposures and health problems, and the effects of treatments.

So far, many articles include the term “real-world” in their titles. The types of research data they cover vary; for example, data from specific hospitals, multicenter studies, patient registries, cohort studies, or annual health check-ups conducted under the law. All data not collected according to a defined schedule and specific clinical setting may be called “RWD.”

Previous reviews have described the status of big and RWD very well [1–3]. This review expands the currently available literature by discussing the usefulness and caveats of RWD collected from annual health checkups and claims data in Japan through recent examples of Japanese research on the association of hypertension with cardiovascular or renal disease. We mainly focused on the Japanese RWD collected from compulsory health check-ups and claims data. Those data are generally collected as electronic records from a wide range of populations through insurers and referred to specifically as “insurer-based RWD” in this review.

## Outline of “RWD”

The definition of RWD varies. The Food and Drug Administration in the U.S. defines RWD as “the data, relating to patient health status and/or the delivery of health care, routinely collected from a variety of sources” [3, 4]. In Europe, the definition is slightly different; RWD is defined as “routinely collected data related to a patient’s health status or the delivery of health care from a variety of sources other than traditional clinical trials” [3, 5]. While definitions from other sources can be found [3], there is a need to unify terminology and recognition, and a unified view of terminology is being considered by the International Council for Harmonization of Technical Requirements for Pharmaceuticals for Human (ICH) and the International Coalition of Medicines Regulatory Authorities (ICMRA); both organizations play crucial roles in the global pharmaceutical landscape [6, 7].

The widespread use of annual health check-ups in Japan has made it easier to obtain large-scale, longitudinal data on the general population. In addition, combining medical information and practices from electronic claims data with health checkup data allows researchers to perform various analyses of health issues. From the perspective of drug regulation, the Good Post-Marketing Study Practice (GPSP), a set of regulations established by the Japanese Ministry of Health, Labour and Welfare (MHLW) to ensure the proper conduct of post-marketing surveillance and studies of pharmaceuticals in Japan, has accepted database research for post-marketing surveillance of pharmaceuticals [8–10]. The GPSP is a regulation aimed at ensuring the quality and integrity of post-marketing studies conducted on pharmaceuticals and medical devices in Japan [8, 9].

## Major available data in Japan

Globally, RWD has been utilized for research on blood pressure (BP); the examples include reports based on the Clinical Practice Research Database (CPRD) [11] and Health Improvement Network (THIN) [12] in the United Kingdom, the Canadian Primary Care Sentinel Surveillance Network in Canada [13], or the System for the Development of Research in Primary Care (SIDIAP) in Spain [14].

As for Japan, representative data sources for the RWD include information from insurance claims, prescriptions, patient registries, and annual health checkups conducted under the law. Electronic health records obtained via wearable devices and apps have also become available in recent years, although the data sources are limited. Of these, the use of large claims and annual health check-up data held electronically by insurers, i.e., insurer-based RWD, has gained attention for academic purposes in Japan.

**Fig. 1:**
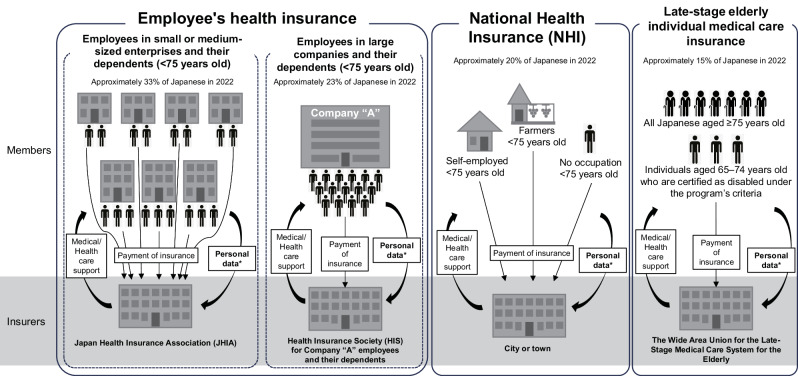
Major health insurance type and outline of the system in Japan. *Insurers receive claims/health screening data through employers or other related organizations. The data is provided to researchers as secondary data for use under appropriate contracts among related organizations

Japan has a universal health insurance system, under which people are obliged to enter one of the insurance systems unless, for example, they are enrolled in the Japanese welfare system. Note that the medical expenses of welfare recipients are covered in full by medical assistance provided by prefectural or city governments. The type of insurance system a Japanese person should be enrolled in depends on his or her occupation. Figure 1 summarizes the relationship between occupation and types of insurance systems. In brief, the major health insurance systems in Japan are as follows: (1) the employee’s insurance managed by the Japan Health Insurance Association (JHIA) with employees of small and medium-sized companies; (2) the employee’s health insurance managed by the Health Insurance Society (HIS), which contains data from Japanese employees and their dependents enrolled in health insurance plans primarily run by large-scale enterprises; (3) the National Health Insurance (NHI) with data from self-employed, unemployed, and retired persons and (4) the medical care system for the elderly in the latter stage of life for Japanese aged ≥75 years and those aged 65‒74 years old who are certified as disabled [15, 16]. The characteristics of the population in studies based on Japanese insurer-based RWD vary according to the type of insurer. For instance, the health check-up data from the HIS include a high proportion of young male workers who are likely to have a stable income [17–20].

The databases from the Japanese insurers that contain annual health check-up data with claims are provided to researchers as secondary data to use under appropriate contracts among relevant organizations and are widely used in reported Japanese studies (Table 1). Private companies managing the data from the insurers are often involved during this provision of RWD to researchers, which determines the name of the Japanese insurer-based RWD. Among the Japanese insurer-based RWD in Table 1, the JMDC database is currently the most used in Japan [21], followed by the DeSC databases; these are commercial databases, for which the data cleaning is usually complete. Meanwhile, the MHLW in Japan created the National Database of Health Insurance Claims and Specific Health Checkups (NDB) to integrate all administrative claims and health checkup data collected under the universal health insurance system. In recent years, nationwide data from JHIA, which covers ~40% of the working-age population in Japan’s working-age population, have become available [22, 23]. In addition, the NDB and JHIA nationwide databases are administrative databases with larger sample sizes and are very different from the commercial databases, particularly in terms of ethical considerations.

**Table 1:** Summary of medical databases that include health check-ups used for academic research in Japan

Database (Provider)	Covered insurance type and features
• JMDC database (JMDC, Inc.) • IQVIA Claims data (IQVIA, Inc.) • CrossFact (INTAGE Real World Inc.)	- Covered insurance: Health insurance society (not all but limited to the associations contracted with each database private company) - Due to the character of the insurers providing data, the data includes a relatively healthy population - Commercial databases^a^
• DeSC database (DeSC Healthcare, Inc.)	- Covered insurance: Health insurance society/ National health insurance/ Late-stage medical care system for the elderly (not all but limited to the associations contracted with the database private company) - It covers the major three types of insurers, resulting in a wide range of age individuals - Commercial databases^a^
• The Japan Health Insurance Association (JHIA) database	- Covered insurance: JHIA - Employees of small and medium-sized companies, which covers ~40% of the working-age population in Japan’s working-age population, have become available - Administrative database^a^
• NDB (The Ministry of Health, Labour and Welfare in Japan)	- Covered Insurance: All insurance - It holds information on receipts and health checkups that can be obtained electronically in Japan, therefore, only the database can be called nationwide. Data maintenance is inadequate and application for use is strictly limited. - The medical assistance receipts for welfare recipients are currently pending construction. - Administrative database^a^

This information was from March 2024. We picked up the main available database, which contains both annual health check-ups and claims data

^a^Data cleaning is usually complete in commercial databases, whereas ethical considerations are stricter in administrative databases than in commercial databases

Other than the insurer-based RWD, Medical Data Vision (MDV) Co. Ltd, provided the MDV database, has also been widely used in Japanese academia and includes information extracted from Diagnosis Procedure Combination (DPC) hospitals in Japan. DPC hospitals are relatively large hospitals that treat a wide range of patients, especially those with severe diseases. The MID-NET® medical information database, which collects patients’ data from multiple Japanese hospitals and codes the data consistently, is a highly reliable clinical database, although it is now mainly used for administrative purposes [24]. Almost all data sources currently available in Japan are listed by the Japanese Society for Pharmacoepidemiology [25].

## The usefulness of the RWD in Japan

This section reviews the usefulness of RWD, especially insurer-based RWD, in Japan by introducing the recent evidence of the association between BP and cardiovascular disease using Japanese RWD.

### Hypertension and cardiovascular disease

Assessment of the stable risk estimates for each detailed BP category requires a large sample size. RWD consists of data from a large number of individuals and can capture traditional, but finely stratified, associations between hypertension and cardiovascular diseases.

Using JMDC database, detailed associations between BP and cardiovascular risks have been assessed in over 2 million individuals without the use of antihypertensive medications [19]. Another study involving ~1 million patients with low cardiovascular risk on antihypertensive treatment from the JHIA database observed that patients exhibited a J-shape association between BP and the risks for cardiovascular diseases [23]. A previous study based on the JMDC database identified 16,159 individuals with isolated diastolic hypertension, which is uncommon among all hypertension types, and demonstrated that isolated diastolic hypertension was associated with 1.28 times higher cardiovascular risk than normotensives [18].

The large sample size using the insurer-based RWD also allowed us to stratify a population according to multiple risk factors. The high risk of cardiovascular disease in individuals with high BP and diabetes compared to others has been assessed using the insurer-based RWD in detail in Japan [26, 27]. Another study demonstrated that the CHA_2_DS_2_-VASc score was associated with stroke risk in patients with atrial fibrillation [28, 29]. Using the IQVIA database, one report confirmed the utility of machine learning algorithms for cardiovascular disease risk prediction [30].

### Hypertension and kidney disease

In Japan, the presence of proteinuria is checked by the dipstick test during health checkups in which healthy individuals participate. Although it is not mandatory, serum creatinine (SCr) levels have also been measured in the Japanese regular health check-ups. Longitudinal data on proteinuria and SCr in Japan are useful for exploring the risk factors for impaired renal function in Asian populations [17, 31].

**Fig. 2:**
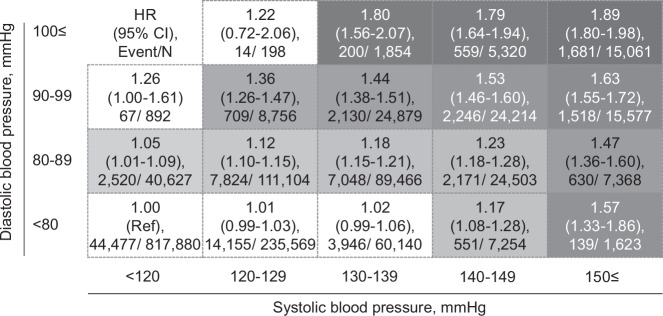
Adjusted hazard ratios (HRs) of CKD according to SBP and DBP. The HRs were adjusted for age, sex, body mass index <18.5 kg/m^2^ and ≥25 kg/m^2^, current smoking status, alcohol consumption, diabetes mellitus, dyslipidemia, and estimated glomerular filtration rate (eGFR) at baseline. The group with systolic/diastolic blood pressure (SBP/DBP) < 120/ ≥ 100 mmHg (*n* = 6) was excluded from the analysis due to the limited number of participants and events. SBP, systolic blood pressure; DBP, diastolic blood pressure; CKD chronic kidney disease, HR hazard ratio, CI confidence interval. This figure was copied from a corresponding article (Suenaga et al. [32])

Our recent study of involving the JMDC database observed 92,587 chronic kidney disease (CKD) events during a mean follow-up of 3.2 years in 1.5 million individuals without CKD, without antihypertensive treatment, and aged <75 years. The detailed cross-classification by both systolic and diastolic BP showed that both isolated high systolic BP and isolated high diastolic BP were strongly associated with CKD risk (Fig. 2) [32]. As a different methodology, the insurer-based RWD can be useful to assess a detailed interaction between systolic BP and other risk factors on CKD risk [20] and to explore the threshold of biomarkers associated with CKD risk [33].

### Effectiveness of antihypertensive treatment

Interventional studies often face limiting factors, collectively known as the “Five Toos”: too few (few cases), too narrow (special patients are excluded), too medium-aged (elderly and children are excluded), too simple (simple method of administration), and too brief (short duration of administration) [34]. RWD is considered to be an approach that can surmount these limitations. Furthermore, while interventional studies focus on the evaluation of “Efficacy,” which is the effect of an intervention under ideal conditions, RWD studies assess “Effectiveness,” which refers to the impact of the intervention in actual clinical or social settings [35]. This distinction highlights the complementary nature of these two types of study designs in understanding the full spectrum of the impact of an intervention [35].

**Fig. 3:**
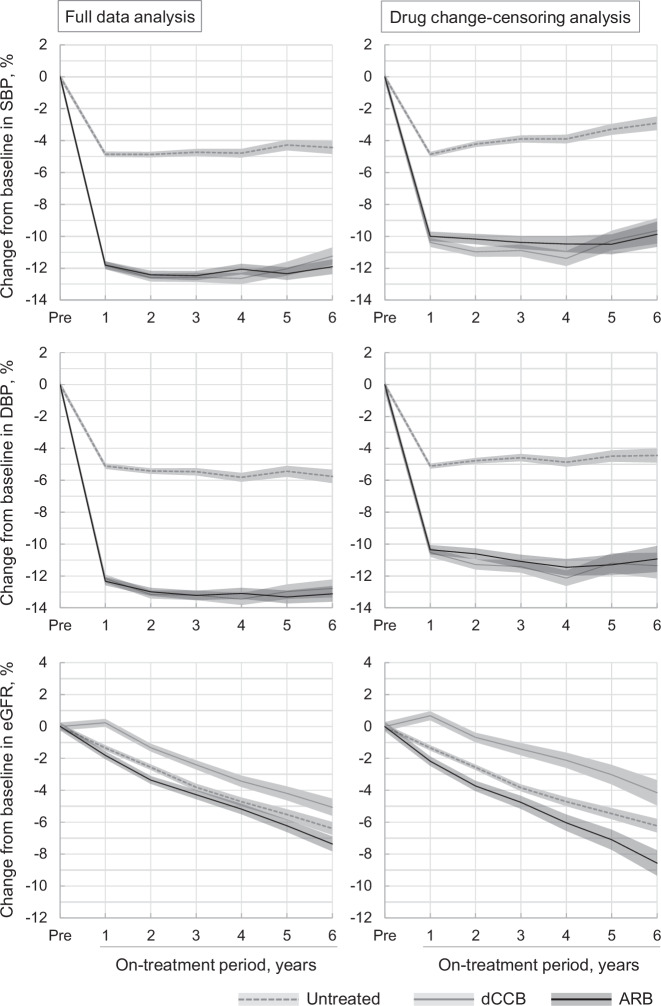
Blood pressure and eGFR change relative to the baseline. ‘Pre’ indicates the pretreatment visit, i.e., baseline. eGFR, estimated glomerular filtration rate. This figure was copied from a corresponding article with permission (Satoh et al. [15]). Use of the material in any format is prohibited without written permission from the publisher, Wolters Kluwer Health, Inc. Please contact permissions@lww.com for further details. The data were based on hypertensive participants (*n* = 10,151), dihydropyridine calcium channel blocker (dCCB) users (*n* = 5078), and angiotensin II receptor blocker (ARB) users (*n* = 5073)

A cohort study based on the RWD in the United Kingdom (CPRD data) reported reductions in BP among 67,274 calcium channel blockers (CCB) new users and 87,440 angiotensin-converting enzyme inhibitors (ACEI)/angiotensin II receptor blockers (ARBs) new-users in primary care setting [11]. Using the Japanese insurer-based RWD, not only a decrease in BP but also a change in eGFR was assessed (Fig. 3) while a suppressed risk of developing proteinuria in new ARB users was also observed [36]. Using a similar methodology, we also quantified the effect of inadequate pharmacological therapy on uncontrolled BP to elucidate the social impact of clinical inertia on uncontrolled hypertension in Japan [37].

### Trends of antihypertensive medications and hypertension prevalence

It is useful to monitor descriptive pharmacoepidemiological data such as prescription trends in clinical practice. However, at a single center, individual prescription preferences can easily influence descriptive data, and unfavorable data may not be published. The insurer-based RWD is generally collected from a wide range of individuals and allows the observation of treatment by various medical institutions. For example, based on the insurer-based RWD data, CCBs seem to be used in a higher proportion of frontline settings and ARBs are commonly preferred over ACEI in Japan [38–40].

One of the major strengths of large-scale RWD is that it captures data from special populations that are difficult to examine in clinical trials. For example, by capturing the medication regimens of pregnant women, it may be possible to overcome the limitations of drug indications among them. A study based on the JMDC database demonstrated that nifedipine was the most commonly prescribed oral antihypertensive medication during pregnancy, followed by methyldopa, hydralazine, and furosemide in 2015 or earlier [41]. Prescription data after 2015 in Japan has also been demonstrated [42, 43].

Since 2014, 95% of claims from hospitals, clinics, and pharmacies have been submitted electronically by all public medical insurers in Japan [44]. While the NDB is the most complete nationwide database in Japan, it is difficult to deal with owing to the large amount of data and researchers must pass the strict screening by the MHLW to use the NDB. Using the NDB, a previous study identified ~27 million Japanese individuals with hypertension and found a regional difference in hypertension prevalence in 2014 [45].

### Impact of social change on BP

Social change, especially through national or governmental interventions, can be considered a grand social experiment. It is a strength of RWD that can reveal the effects of social change on the BP of residents. Another strength of RWD is the speed of data provision, which makes it possible to quickly report the impact of social change.

Japanese hypertension guidelines (JSH) were updated from JSH2014 to JSH2019 [46] in 2019. In the next year of 2020, the first state of emergency due to the coronavirus disease 2019 (COVID-19) pandemic was declared. Using DeSC database, we observed that systolic BP specifically decreased by ≤1 mmHg in fiscal year 2019 among the treated participants, suggesting the limited impact of the JSH guideline update. Meanwhile, systolic/diastolic BP increased by 1–2/ 0.5–1 mmHg in fiscal year 2020 in Japan [15]. A similar increase in BP during the COVID-19 pandemic was also reported among adults in the U.S. [47, 48].

Based on the NDB Open Data, which is the summarized nation-wide data, are available to the public [49]. Using the NDB Open Data, we confirmed that antihypertensive prescriptions were adequately maintained throughout the COVID-19 pandemic in Japan, despite the temporal concerns raised about the potential adverse effects of ARBs and ACEIs on patients with COVID-19 in 2020 [50]. However, the NDB Open Data only provides information on the number of tablets prescribed in each fiscal year and offers data solely on the 100 most prescribed drug products within each therapeutic category [50]. By improving these aspects, researchers would be able to track drug prescriptions or disease trends easily using NDB Open data.

## Caveats of analyses using RWD

Despite the usefulness of RWD collected as electronic records from a wide range of populations, we must be cautious about several points. Here, we focused on issues found in the use of Japanese insurer-based RWD; while the same considerations can also be applied to countries other than Japan, as long as the data extracted is in a similar manner to the Japanese insurer-based RWD.

### Population uncertainty

Removing identifiers, i.e., de-identification, reduces privacy risks to individuals and is globally required for the secondary use of RWD [51, 52]. Moreover, in Japan, data providers are required to anonymize data when they offer it to researchers according to the Personal Information Protection Law in Japan [53]. Most details regarding the basic characteristics of the population, including geographical information or the type of work, are basically unclear in Japanese insurer-based RWD, except for the NDB and JHIA nationwide database. Traditional prospective cohort studies in the general population can consider regional characteristics. Furthermore, based on the Industrial Safety and Health Act, the annual health check-up is mandatory among employees in Japan [54]. Meanwhile, since a health check-up is only recommended, rather than mandatory, to others, health check-up data of non-employed individuals may include a higher selection bias than those of employees.

Confirming whether the data are representative and generalizable to the relevant population is important [55, 56]. We must keep in mind that a large sample size does not overcome the problems of systematic error but only allows us to suppress random error, stratify participants by fine categories, or include many covariates in a statistical model. Statistical methods, such as weighting the data to a targeted population, can reduce systematic errors but may not eliminate them.

### Limited validity of outcomes

The validity of outcomes remains a global issue in studies that define outcomes based on electronic health data, especially claims data [57, 58]. While cardiovascular diseases are generally defined based on disease codes or disease names in claims records, the disease codes for outcome definitions considerably vary among studies and countries [59]. In this context, although validation studies have been performed worldwide [60–63], the external validity of individual results does not extend across countries.

Clinicians register the disease name or codes on the claims to receive reimbursement, which mainly causes inaccurate input of disease codes in claims data [64]. When using clinician-assigned disease codes as cardiovascular outcomes, we should acknowledge that the outcome is not a true endpoint but only a proxy endpoint. Several studies have merely examined the consistency of definitions within the same database [65, 66], and these studies are often cited as evidence indicating that the outcome definitions based on claims data are valid. However, the gold standard in outcome validation studies should be based on specialists’ assessments using medical records with objective measures in clinical practice.

To calculate the relative risk with no bias, the outcome definition must reveal perfect specificity and nondifferential sensitivity independent of exposure [67–70]. In the validation of outcomes based on claims, the positive predictive value (PPV) is generally surveyed because of the difficulty in determining the specificity of the outcome. High sensitivity of the outcome is also required for the calculation of absolute risk, as well as for calculating accurate relative risk.

**Fig. 4:**
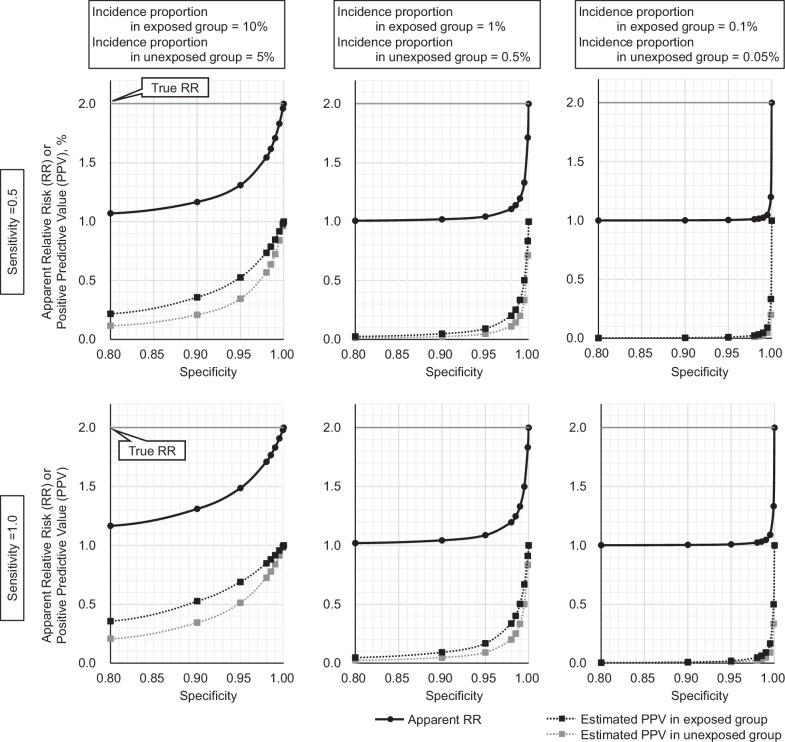
Apparent relative risks and positive predictive values according to specificity and incidence rate in a simulation. The *y*-axis is for the relative risk (RR) and positive predictive value (PPV). The curve is based on a power function

We simulated the association between the specificity of the outcome and the apparent relative risk (aRR), as shown in Fig. 4. If the specificity is low, the relative risk may be underestimated. Furthermore, in situations with a low incidence proportion, high specificity is required to accurately estimate relative risk. We assumed that the incidence proportions are 0.1% and 0.05% in the exposed and unexposed groups, respectively, and the sensitivity of the outcome was 0.5 (50%); the true relative risk of exposure is 2.0. Under this setting, even if the specificity of the outcome definition was 99.90% (the estimated PPVs of 20.0% in the unexposed group and 33.4% in the exposed group, respectively), the aRR was 1.20. The aRR increased to 1.712 by increasing the specificity from 99.90% to 99.990%, which corresponds to an estimated PPVs of 71.4% and 83.3% in the unexposed and exposed groups, respectively.

The required PPV and specificity can be calculated using target aRR, proportion in exposed group or in unexposed group [Pe or Pue], and sensitivity [SENS] as follows:

$$	{{\bf{R}}}{{\bf{e}}}{{\bf{q}}}{{\bf{u}}}{{\bf{i}}}{{\bf{r}}}{{\bf{e}}}{{\bf{d}}}\,{{\bf{s}}}{{\bf{p}}}{{\bf{e}}}{{\bf{c}}}{{\bf{i}}}{{\bf{f}}}{{\bf{i}}}{{\bf{s}}}{{\bf{i}}}{{\bf{t}}}{{\bf{y}}}[{{\bf{r}}}{{\bf{S}}}{{\bf{P}}}{{\bf{E}}}{{\bf{C}}}]\\ 	 =\frac{{{\rm{aRR}}}\times {{\rm{Pue}}}\times (1-{{\rm{SENS}}})-{{\rm{aRR}}}+{{\rm{Pe}}}\times ({{\rm{SENS}}}-1)+1}{{{\rm{aRR}}}\times ({{\rm{Pue}}}-1)-{{\rm{Pe}}}+1}$$

$$	{{\bf{R}}}{{\bf{e}}}{{\bf{q}}}{{\bf{u}}}{{\bf{i}}}{{\bf{r}}}{{\bf{e}}}{{\bf{d}}}\,{{\bf{P}}}{{\bf{P}}}{{\bf{V}}}\,{{\bf{i}}}{{\bf{n}}}\,{{\bf{e}}}{{\bf{x}}}{{\bf{p}}}{{\bf{o}}}{{\bf{s}}}{{\bf{e}}}{{\bf{d}}}\,{{\bf{g}}}{{\bf{r}}}{{\bf{o}}}{{\bf{u}}}{{\bf{p}}}\\ 	 =\frac{{{\rm{SENS}}}\times {{\rm{Pe}}}}{{{\rm{SENS}}}\times {{\rm{Pe}}}+(1- \, {{\rm{Pe}}})\times (1- \,{{\rm{rSPEC}}})}$$

Table 2 summarizes the examples of required specificity and PPV values based on these equations. The required PPV is highly dependent on the acceptable aRR [57]. If the acceptable aRR is set at 1.8 in the situation where the true relative risk is 2.0, the required PPV is ~80%. Although there are no clear criteria, a PPV of less than 50% would not be acceptable for calculating the true relative risk.

**Table 2:** Required specificity and PPV values according to apparent relative risks, sensitivity, and incidence proportion

Fixed parameters				Required values		
	Incidence proportion				PPV	
Apparent relative risk	Non-exposure group	Exposure group	Sensitivity in both groups	Specificity in both groups	Non-exposure group	Exposure group
1.2	5.0%	10.0%	50%	91.667%	24.00%	40.00%
1.2	0.50%	1.0%	50%	99.020%	20.40%	34.00%
1.2	0.050%	0.10%	50%	99.900%	20.04%	33.40%
1.2	0.0050%	0.010%	50%	99.990%	20.00%	33.34%
1.2	5.0%	10.0%	100%	83.333%	24.00%	40.00%
1.2	0.50%	1.0%	100%	98.039%	20.40%	34.00%
1.2	0.050%	0.10%	100%	99.800%	20.04%	33.40%
1.2	0.0050%	0.010%	100%	99.980%	20.00%	33.34%
1.5	5.0%	10.0%	50%	97.619%	52.50%	70.00%
1.5	0.50%	1.0%	50%	99.751%	50.25%	67.00%
1.5	0.050%	0.10%	50%	99.975%	50.03%	66.70%
1.5	0.0050%	0.010%	50%	99.998%	50.00%	66.67%
1.5	5.0%	10.0%	100%	95.238%	52.50%	70.00%
1.5	0.50%	1.0%	100%	99.502%	50.25%	67.00%
1.5	0.050%	0.10%	100%	99.950%	50.03%	66.70%
1.5	0.0050%	0.010%	100%	99.995%	50.00%	66.67%
1.8	5.0%	10.0%	50%	99.383%	81.00%	90.00%
1.8	0.50%	1.0%	50%	99.938%	80.10%	89.00%
1.8	0.050%	0.10%	50%	99.994%	80.01%	88.90%
1.8	0.0050%	0.010%	50%	99.999%	80.00%	88.89%
1.8	5.0%	10.0%	100%	98.765%	81.00%	90.00%
1.8	0.50%	1.0%	100%	99.875%	80.10%	89.00%
1.8	0.050%	0.10%	100%	99.988%	80.01%	88.90%
1.8	0.0050%	0.010%	100%	99.999%	80.00%	88.89%

In all lines, the true relative risk is 2.0

*PPV* positive predictive value

The cardiovascular disease outcomes based on disease codes admitted by clinicians, without even procedure codes, have been often used in many studies including the abovementioned studies [18, 19, 27]. However, the validity of outcomes based only on the International Classification of Diseases (ICD) codes and disease names in Japanese claims is unfortunately unreliable for defining cardiovascular diseases [71]. To deal with this issue, some studies have combined the corresponding ICD codes with hospitalization or treatment codes when defining [23, 28, 29].

The PPVs defined only by diagnostic codes were low, especially for heart failure and stroke (Table 3) [72–74], suggesting low specificity for these outcomes. In this regard, the relative risk estimate based on the ICD-10 codes is assumed to be lower than the true relative risk. Several methods have been suggested to increase the PPV of cardiovascular outcome definitions based on claims data (Table 3). It should also be considered that the sensitivity and specificity of disease definitions based on claims differ among institutions and databases [75].

**Table 3:** Reported PPV of cardiovascular outcome definitions based on codes of medical claim

Outcome name	Report	Definition	PPV
Acute myocardial infarction	Nakai et al. [72]	ICD-10 code (I21)	0.79 (0.78–0.80)
Improved definition^a^	Nakai et al. [72]	ICD-10 code + emergency hospitalization only	0.85 (0.84–0.86)
Acute myocardial infarction	Shima et al. [74]	ICD-10 codes (I21, I22)	0.82 (0.73, 0.89)
Improved definition^a^	Shima et al. [74]	ICD-10 codes + Drugs	0.87 (78.3, 0.93)
Acute coronary syndrome	Kanaoka et al. [71]	ICD-10 codes (I20.0, I21, I22, I23)	0.35 (0.32–0.37)
Improved definition^a^	Kanaoka et al. [71]	ICD-10 codes + PCI	1.00 (0.99–1.00)
Coronary artery disease	Fujihara et al. [73]	ICD-10 codes (I20, I21, I22, I23, I24, I25)	0.39 (0.32–0.43)
Improved definition^a^	Fujihara et al. [73]	DPC codes	1.00 (0.93–1.00)
Acute Heart Failure	Nakai et al. [72]	ICD-10 code (I50)	0.57 (0.56–0.58)
Improved definition^a^	Nakai et al. [72]	ICD-10 code + additional disease codes from DPC + emergency hospitalization	0.83 (0.82–0.84)
Acute Heart Failure	Kanaoka et al. [71]	ICD-10 codes (I50, I11.0)	0.15 (0.14–0.16)
Improved definition^a^	Kanaoka et al. [71]	Original algorithm	0.79 (0.76–0.83)
Acute Heart Failure	Fujihara et al. [73]	ICD-10 codes (I50, I11.0, I13.0, I13.2)	0.10 (0.08–0.01)
Improved definition^a^	Fujihara et al. [73]	DPC and/or ICD-10 codes + medication	1.00 (0.85–1.00)
Hemorrhagic stroke	Shima et al. [74]	ICD-10 codes (I60, I61, I62)	0.46 (0.34, 0.57)
Improved definition^a^	Shima et al. [74]	ICD-10 codes + Medical procedures	0.46 (0.35, 0.58)
Non-traumatic intracerebral hemorrhage	Fujihara et al. [73]	ICD-10 codes (I60, I61, I62)	0.53 (0.39–0.53)
Improved definition^a^	Fujihara et al. [73]	DPC codes	1.00 (0.81–1.00)
Ischemic stroke	Shima et al. [74]	ICD-10 codes (I63)	0.31 (0.23, 0.40)
Improved definition^a^	Shima et al. [74]	ICD-10 codes + drugs + medical procedures	0.44 (0.33, 0.57)
Ischemic stroke	Fujihara et al. [73]	ICD-10 codes (G459, I63)	0.53 (0.35–0.64)
Improved definition^a^	Fujihara et al. [73]	DPC codes	1.00 (0.82–1.00)

We extracted the PPV of the outcomes based only on the ICD-10 codes in each report

^a^We extracted the definitions with the highest PPV for each report

*PPV* positive predictive value, *ICD-10* International Classification of Diseases, Tenth Revision, *DPC* diagnosis procedure combination

**Fig. 5:**
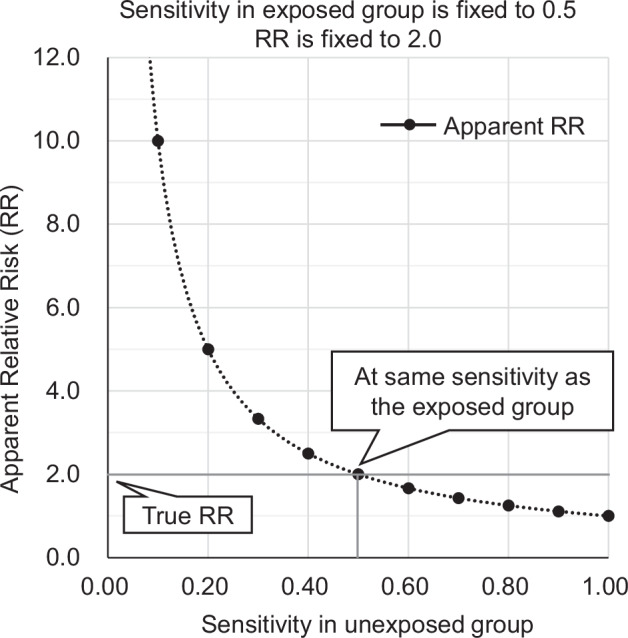
Apparent relative risk according to sensitivity in the unexposed group in a simulation. The results indicate the apparent relative risk under the assumption that the sensitivity differed between the exposed and unexposed groups, whereas the specificity is 1.0 (perfect) in both groups. The apparent relative risk was not affected by the incidence rate or specificity. The curve is based on a rational function

We simulated the change in the aRR by changing sensitivity. If the sensitivity of the outcome is lower in the non-exposed group than in the exposed group, the relative risk may have been overestimated (Fig. 5). For example, it can be assumed that non-hypertensive patients who have not visited a medical institution have fewer opportunities to have disease codes in the claim data. This can result in a lower sensitivity of the outcome based on the disease code in non-hypertensive patients (non-exposed group) than in the hypertensive group (exposed group); the aRR can be higher than the true relative risk. Therefore, disease definition based on disease codes alone should be used with caution when estimating relative risks unless the corresponding disease code has been validated.

### Limited accuracy of BP

The limited accuracy of BP may be a problem specific to the Japanese insurer-based RWD. Annual health check-up data from the insurer-based database were obtained from a real practice environment. Health checkups in Japan are encouraged to comply with the guidelines recommended by the Japanese MHLW [76]. The same method as that in the JSH guidelines is recommended to measure BP, that is, twice consecutively in the sitting position for annual health check-ups [46]. However, actual management depends on each responsible organization that carries out health checkups. Adherence to the recommendations may be limited in a busy practice setting. Because a single measurement for BP is accepted in case of a situation [76], the BP data may include the single-measured BP, suggesting the limited accuracy and substantial random errors in the BP measured in Japanese health check-ups.

According to a Japanese survey on the actual status of BP measurement at health check-ups in 2019, only 19% of the health check-up organizations routinely perform two measurements; 68.8% of the centers perform a second measurement only if the first measurement result is high, and 34.4% of the centers use the lower value among multiple measurements [77]. Even in clinical practice, ~50% of Japanese general practitioners answered that BP is measured only once at their office [78].

The random errors in BP may be covered by a large sample size, which is a strength of RWD. However, we must be careful of the regression-to-mean effect of BP, especially when participants were stratified by BP. As we can see in Fig. 3, in the untreated group extracted according to the definition of BP ≥ 140/ ≥ 90 mmHg, BP dropped by 5% in the year following baseline, exactly representing the regression-to-mean effect. In addition, BP data from health checkups are systematically biased owing to the white-coat effect. The difference in measurement BP devices may also influence the validity of the BP. Although this problem on BP accuracy is not unique to RWD research, improvements in BP measurement in Japanese health checkups are highly recommended to reduce the risk of misdiagnosis.

### Issues in traceability to follow-up

In Japan, the insurer-based database contains electronic claims data issued at the time of an individual’s visit to a medical institution. This means that all electronic claims information can be collected regardless of the medical facility that the individual visits. However, the insurer-based database links individuals mostly by their insured person numbers, which alter with a change in insurer affiliation due to a job change or retirement, thereby leading to loss of track of the individuals. Although frequent job changes may not be common in Japan than in other countries, we should be cautious about this point.

Because of the Japanese law regarding health insurance (Fig. 1), Japanese individuals are forced to change their health insurance to the late-stage elderly individual medical care insurance when they reach the age of 75. This means that there is an absolute end-of-tracking point at age 75 in most Japanese insurer-based databases. Almost all results introduced in the “The usefulness of the RWD in Japan” section except for those using NDB have this problem [15, 17–20, 23, 28, 29, 31–33, 36, 38–42].

### Other biases including indication and immortal biases

RWD have other biases owing to their nature [79]. When comparing the effects of treatments, indication bias can be a common issue caused by the assignment of patients to different treatments based on their symptoms or medical conditions, potentially skewing the results and interpretation of treatment outcomes. Observational studies have reported higher cardiovascular risk in individuals receiving antihypertensive treatments than in untreated individuals [80, 81]. This phenomenon is considered to be caused by “indication bias” because there is a considerable gap in the backgrounds between individuals with treatment and those without [82]. Balancing confounding factors between groups, such as through propensity score matching, is effective in reducing indication bias. However, methods utilizing propensity scores only balance confounding factors between groups and cannot eliminate the indication bias.

Even if the gap in background factors can be overcome, there may be immortal bias in the comparison between the untreated and new treatment user groups [79]. Immortal bias is caused because new users must live until the time of treatment initiation after baseline. The new users tend to have less time and opportunity to encounter any events than the untreated group, resulting in a lower risk for the new users. Although there are several statistical methods to minimize this problem, it is difficult to eliminate it completely [79]. The simplest solution is to make both groups new users of different treatments, which is called as a new-user design [79].

### Technical problems

RWD is essentially big data containing multiple individual records. Constructing a dataset for analysis is time-consuming. Although the development of the usage of the NDB may allow us to control the data from entire Japanese population, it must be difficult to deal with it even with modern computers. It may not be necessary to handle all the data if the representativeness of the population is ensured. In the future, data handling must be streamlined by specialists such as data scientists.

## Conclusions

Despite the aforementioned limitations, the use of RWD collected electronically on a large scale continues to be promoted. In addition, each insurer has recently been required to operate its health services based on a “data health plan” that implements efficient and effective health services using a Plan-Do-Check-Act cycle based on data analysis of medical costs, health checkups, and other data [83]. This is not only to measure the contribution of BP to health problems in the real world and the “effectiveness” of treatment but also to observe populations that are not included in clinical trials and outcomes that are not fully expected. With appropriate analysis, the results can be similar to those obtained from clinical trials and previously proven pharmacological phenomena in the real world [36]. In addition, the reliability of the RWD should be improved. For example, common good algorithms for outcome definitions based on claims data and appropriate BP measurements during annual health checkups should be promoted. Because claims information can be used to solve social problems, it may be needed to develop a system based on the assumption that it will also be used for scientific analyses. The use of RWD is expected to improve public health and medical care.
